# Toward a ‘Holistic’ Approach to Promoting Physical and Mental Health in Children and Adolescents Through Physical Activity

**DOI:** 10.1111/apa.70678

**Published:** 2026-07-14

**Authors:** Chloe Emonson, Nicole Rinehart, Caterina Pesce, Carlo Agostoni, Livio Luzi, Spyridoula Vazou

**Affiliations:** ^1^ School of Psychological Sciences, Faculty of Medicine, Nursing and Health Sciences Monash University Clayton Victoria Australia; ^2^ Department of Kinesiology Michigan State University East Lansing Michigan USA; ^3^ Pediatric Unit Fondazione IRCCS Ca' Granda Ospedale Maggiore Policlinico Milan Italy; ^4^ University of Milano Milan Italy; ^5^ IRCCS MultiMedica Milan Italy

**Keywords:** health promotion, holistic health, implementation, physical activity, school, transdisciplinarity

## Abstract

**Aim:**

Physical activity in childhood and adolescence is a cornerstone of holistic health, supporting physical growth, cognitive development, emotional wellbeing and social connectedness. We aimed to explore how lifestyle‐based physical activity interventions in enriched school and community settings can support development and address health inequities.

**Methods:**

This paper is a narrative mini‐review that discusses the promotion of physical activity for holistic development in the light of a selection of representative and scalable whole‐school programs that incorporate transdisciplinary initiatives. In the framework of such scalable programs, we highlight the role of collaborative governance and public–private partnerships, including corporate social responsibility commitments.

**Results and Conclusion:**

Comprehensive School Physical Activity Programs and Whole School, Whole Community, Whole Child frameworks illustrate the potential of physical activity as an affordable, equitable strategy for promoting children's and adolescents' healthy development. Globally, despite growing evidence of effectiveness, systemic barriers continue to impede the timely translation of scientific evidence into practice and the potential of policy and practice to orient scientific research. To address this gap, new implementation approaches such as the dual‐logic framework that integrates scientific rigour with policy‐driven pragmatism can play a crucial role in creating sustainable pathways to implement lifestyle‐based interventions at scale.

## Summary

- Despite evidence that physical activity supports holistic health development, strategies are generally siloed, and translation gaps limit delivery of effective interventions for children and youth.
- We highlight that whole‐school, transdisciplinary programs, supported by partnerships, can be scalable across school and community settings toward health‐enhancing physical activity participation.
- Implementation frameworks, such as dual‐logic approaches, may accelerate translation into sustainable and scalable pathways for promoting physical activity and positive health trajectories.

## Graphical abstract

Moving beyond siloed physical activity interventions toward holistic approaches can best support children's healthy developmental trajectories.

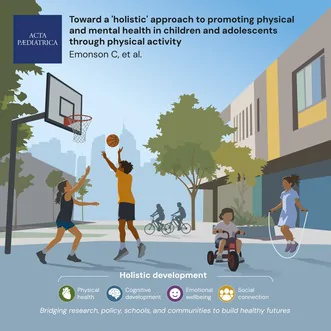

**Abbreviation**
WHO: World Health Organization

## 1 Introduction and Context

Supranational agencies are committed toward the promotion of optimal physical growth and mental development. The World Health Organization [1, p. 1] defines health as ‘a state of complete physical, mental and social wellbeing and not merely the absence of disease or infirmity’. Indeed, this internationally recognized interpretation emphasizes that all dimensions of wellbeing—physical, mental and social—are equally important for achieving health, which is a dynamic, multidimensional construct shaped by a range of behavioural and environmental influences that should be accounted for using a combined approach [2]. Thus, health promotion should provide opportunities to develop physical, mental and social assets, not only medical solutions.

Physical inactivity is one of several lifestyle factors that are leading risks for mortality globally [3] and are therefore fundamental in health promotion. Unfortunately, adverse lifestyle exposures—including physical inactivity—disproportionally impact minority and vulnerable populations, contributing to health inequities [4]. However, from a medical perspective, hereditary traits can interact with positive lifestyle factors such as increased physical activity under changing environmental conditions, leading to an advantageous change from unfavourable transmissible epigenetic alterations [5].

Given the relationship between physical activity and positive health outcomes, national and supranational health organizations, such as the WHO, have developed guidelines outlining the recommended levels of physical activity that people across the lifespan, as early as infancy, should engage in. Table 1 provides an overview of the WHO guidelines for physical activity and sedentary behaviour for children under 5 years of age [6] and for the broader population from 5 years up to old age [7]. For children and adolescents, most national physical activity guidelines converge on the ‘gold standard’ of at least 60 min of moderate‐to‐vigorous physical activity, even though there is large cross‐country variability in the quality of national guidelines and in their rigour in referring to a solid evidence base [8].

**TABLE 1:** WHO guidelines for physical activity and sedentary behaviours from infancy to older adulthood.

Age group	Physical activity	Sedentary screen time
Infants (less than 1 year)	At least 30 min a day spent in a variety of ways, particularly through interactive floor‐based play	Not recommended (0 min). When sedentary, engaging in reading and storytelling
Young children (1–2 years)	At least 180 min a day spent in a variety of types at any intensity, including moderate‐to‐vigorous intensity	Not recommended (0 min) for 1‐year old; no more than 60 min for 2‐year old. When sedentary, engaging in reading and storytelling
Young children (3–4 years)	At least 180 min a day spent in a variety of types at any intensity, of which at least 60 min moderate‐to‐vigorous intensity	No more than 1 h (less is better). When sedentary, engaging in reading and storytelling
Children and adolescents (5–17 years)	At least 60 min a day of moderate‐to‐vigorous intensity, including muscle‐ and bone‐strengthening activities at least 3 days a week	The amount of daily time spent being sedentary, particularly recreational screen time, should be limited
Young and middle‐aged adults (18–64 years)	At least (a) 150–300 min of moderate‐intensity aerobic; or (b) 75–150 min of vigorous‐intensity aerobic or (c) an equivalent combination throughout the week, including muscle‐strengthening activities at least 2 days a week	The amount of daily time spent being sedentary should be limited and replaced with physical activity of any (including light) intensity
Older adults (65 years and older)	At least (a) 150–300 min of moderate‐intensity aerobic; or (b) 75–150 min of vigorous‐intensity aerobic or (c) an equivalent combination throughout the week, including (i) muscle‐strengthening activities at least 2 days a week or (ii) varied multicomponent activities that emphasize functional balance	The amount of daily time spent being sedentary should be limited and replaced with physical activity of any (including light) intensity

Importantly, countries are increasingly expanding their focus on overall movement behaviours during a 24‐h period and the proportion of physical activity, sedentary screen time and sleep within the 24‐h frame, rather than on structured physical activity training only [9]. In school settings, this means expanding the focus from curricular physical education only to programs that provide enhanced movement opportunities throughout the entire school day [10]. Physical activity interventions embedded within the school environments offer a promising pathway to support young people to meet physical activity recommendations and promote whole‐child development to reduce unmet physical and mental health needs. By leveraging an everyday setting and providing enjoyable and enabling physical activity experiences, schools also present a scalable opportunity to build lifelong healthy behaviours and mitigate health inequities [11].

Despite the number of studies demonstrating efficacy, several issues must be addressed to prevent ineffective translation of physical activity interventions into practice and ensure widespread implementation, scale up and maintenance [12]. First, the current emphasis on a hierarchy of evidence (i.e., conducting pilot trials before efficacy trials before effectiveness trials before implementation) hinders widespread implementation before a program has moved through the full evidence pipeline. Second, a lack of transferability and adaptability of programs that are not informed by a systems thinking perspective on the barriers and facilitators of real‐world circumstances during program development can hinder successful scale‐up. Third, a lack of buy‐in from stakeholders can reduce scalability, as quality‐sustained partnerships are required for widespread implementation in real‐world settings and should be established in the earliest phase [12].

Thus, to support successful development and implementation of school‐based physical activity programs, transdisciplinary and systems‐based approaches are needed to ensure program relevance, feasibility and sustainability. Indeed, a systematic review by Cassar et al. [13] identified facilitators of the adoption, implementation and sustainability of school‐based physical activity interventions, including co‐involvement in the early stages, shared decision making, involvement of experts across disciplines (sports, health and education) and continued participation of all partners. Additionally, cross‐sector partnerships are needed to support program sustainability by encouraging initiatives to be embedded into school systems rather than delivered as short‐term add‐ons [14].

The following sections discuss the potential for physical activity to benefit health holistically, the role of transdisciplinary approaches, whole‐school models and cross‐sector partnerships in enabling programs that serve as a foundation for whole‐child development, and provide examples of programs in practice globally to foster equitable and scalable health promotion for children.

## 2 A Holistic Perspective on Outcomes: Interconnectedness of Physical Activity Benefits in Multiple Domains

This paper advocates for the use of school‐based physical activity programs that foster holistic health development to reduce unmet physical and mental health needs in childhood and support positive lifelong outcomes. Beyond conceiving physical activity as a sole means to increase energy expenditure, the wide‐ranging health benefits of physical activity are encapsulated by comprehensive models such as the Human Capital Model [15]. This model conceptualizes physical activity as a lifestyle‐based affordable investment in human capital that is fundamental to health across the multidimensional context of development including intellectual, financial, physical, emotional, individual and social domains.

Despite agreement on the multidimensional aspects of health—as outlined by the WHO [7]—and the well‐established role of physical activity in promoting a myriad of health benefits across domains—as outlined by the Human Capital Model [15], attempts to leverage physical activity to positively impact children's health and developmental outcomes are typically siloed. Indeed, strategies to promote physical activity are generally approached in a discipline‐specific manner and applied only to an individual developmental domain. Regarding children and adolescents, interventions focused on the physical domain mainly target physical fitness, cardiometabolic health and adiposity [16]; those focused on the cognitive domain mainly target higher‐level cognition—executive function—relevant to health and wealth [17]; and those focused on the social–emotional domain mainly target mental health and especially its ill‐being dimension such as depression and anxiety [18].

The siloed nature of the promotion of physical and mental health that still dominates interventional research and praxis is in contrast to the increasing evidence that their development is intertwined [11]. A growing body of motor developmental research has raised awareness that motor competence is central to positive trajectories of health, with a flourishing of research on the interrelations of motor development with physical health (e.g., fitness, weight status) [19], cognitive health and socio‐emotional mental health [20]. Moreover, it is important to note that motor competence is central to physical literacy, a construct that has expanded beyond sole competence to a holistic level encompassing physical, mental and social health, with significant implications for practice and policy [21]. In this light, it becomes increasingly relevant to consider physical activity not merely as a necessary means of energy expenditure, but as an ‘enriched’ environment intentionally designed by experts for the active participation and healthy development of physically literate children and adolescents.

This evidence shifts the focus of health‐enhancing physical activity for children and adolescents from a mere quantitative dosage required to ensure physical health [7] to qualitative intervention characteristics designed to promote motor competence and physical literacy that can range from free play to more deliberate forms of play and structured practice and span from early childhood to adulthood [22].

## 3 A Holistic Perspective on Interventions: Transdisciplinary Approaches to Comprehensive Physical Activity Programs

Health‐promoting initiatives are also strengthened by the essential role of transdisciplinary collaboration. Transdisciplinary approaches provide a comprehensive framework for understanding factors that influence human health by promoting collaboration, sharing responsibility and integrating concepts, theories and methods across disciplines to generate insights that cannot be produced within a single field [23]. These approaches are especially needed, for example, in the treatment of children and adolescents experiencing health inequities such as obesity, which requires the joint involvement of medical professionals, dieticians, paediatric psychologists, physiotherapists and/or experts in physical activity [24]. However, changing physical activity behaviour may be unrealistic and potentially discouraging for many individuals. The feelings and memories of physical activity in the school environment represent a key determinant of future physical activity levels [25]. Thus, schools are a privileged context where physical activity promotion can and should be approached in an affordable, enjoyable and acceptable fashion. Moreover, schools are well‐positioned to overcome barriers that arise due to social inequities by reaching a large majority of young people and their families [26]. This is especially important in light of the ‘treatment gap’, which occurs when more people experience a health condition than receive appropriate care services. This is the case, for example, of youth mental health, where there have been several calls for the need to extend the provision of care and enhance the scalability and reach of support [27].

In schools, mental health support is commonly delivered through multi‐tiered support systems—a three‐tier framework for providing foundational and targeted programs—and can incorporate physical activity initiatives [11, 28]. The most common types of multi‐tiered support systems include school‐wide positive behaviour supports and social and emotional learning programs. Whereas Tiers 2 and 3 provide targeted and intensive interventions to address the needs of a small number of at‐risk or high‐need students, respectively, Tier 1 initiatives are provided to all students and focus on preventive, school‐wide practices [28]. Thus, physical activity programs that foster holistic healthy development and are delivered as a Tier 1 initiative have the potential to reduce the number of children experiencing physical and mental health difficulties with unmet needs and increase the number of children who develop into thriving adults [11].

However, as indicated previously, current physical activity initiatives generally do not consider the interconnectedness of physical activity outcomes in multiple domains. Thus, the established multi‐tiered support systems structure should be expanded to foster holistic development using physical activity programs, for example by expanding (a) teams to incorporate varied expertise (e.g., classroom teachers, school psychologists, physical educators), (b) student screening processes to consider physical, cognitive and psychological attributes and (c) evidence‐based practices to include approaches that target several areas of development [11]. In practice, expanding the multi‐tiered support systems structure could involve providing both meaningful physical activity opportunities universally to all students (Tier 1) and targeted physical activity interventions to children who have been identified as experiencing physical, cognitive or psychosocial difficulty (Tiers 2–3).

In this regard, an impactful framework that has informed practice and policy development in line with the Human Capital Model's recognition of physical activity as an investment for achieving holistic health and development is the Comprehensive School Physical Activity Program [10]. This framework encompasses, along with formal physical education, other opportunities for students to be active during school (e.g., physical activity during recess, classroom‐based physical activities like active breaks and physically active learning); before and after school (e.g., active commuting to school, afterschool sports training); and through staff involvement as well as family and community engagement. In doing so, Comprehensive School Physical Activity Programs provide concrete opportunities to translate population‐level models of health and human capital and multi‐tiered support system initiatives into efficacious school‐based practices that foster positive youth development through multicomponent, quality experiences of physical activity [29].

Importantly, Comprehensive School Physical Activity Programs align closely with the Whole School, Whole Community, Whole Child framework that places whole‐child development at the center of the education system and adopts an inherently holistic approach by encompassing health education; counselling, psychological and social services; community and family engagement and physical education and physical activity [30]. Whereas Comprehensive School Physical Activity Programs emphasize physical activity as a key lever within schools, the Whole School, Whole Community, Whole Child framework provides a broader organizing framework that recognizes schools as complex, adaptive systems that require coordinated action across multiple stakeholders including researchers, partner school specialists and philanthropic organizations. Together, these frameworks move beyond singular and discipline‐specific approaches and support holistic health promotion through physical activity [31].

## 4 A Holistic Perspective on Evidence‐to‐Policy Transfer: Public‐Private Partnerships and a ‘Dual‐Model’ Logic in Scaling Physical Activity Programs

In recent years, there has been an increase in school‐based approaches to physical activity promotion that entail holistic features, as highlighted in a recent conference dedicated to whole‐school approaches to physical activity promotion (Whole School Physical Activity [WSPA] 2024 conference; https://www.wspa2024.co.uk/conference‐programme‐at‐a‐glance). The programs selected for the WSPA conference include diverse contexts: Australia (‘TransformUs!’) [32], Estonia (‘Schools in Motion’) [33], Finland (‘LIKES Schools on the Move’) [34], Italy (‘Joy of Moving: JoM’) [35], Norway (‘SEFAL Centre for Physically Active Learning’) [36], the UK (‘Creating Active Schools’) [37] and the USA (‘SWITCH Program’) [38].

The above programs are representative of a broader range of valuable, large‐scale multicomponent initiatives developed in the school setting which demonstrate the ability to impact child and adolescent health (e.g., the Healthy School Start Study) [39, 40] but also the need to address issues that emerge when it comes to implementing pragmatic trials at scale (e.g., the ACTNOW study) [41]. All these initiatives are examples of how school‐based comprehensive physical activity programs can have holistic characteristics at different levels, targeting multiple outcomes by means of multicomponent interventions implemented within a multi‐stakeholder system. Indeed, across these initiatives, following shared features emerge: (1) a commitment to advancing multiple dimensions of child development (physical, cognitive, social and emotional) in a unified manner; (2) implementation and scale‐up through collaboration involving education, health and community stakeholders and (3) transdisciplinary design and implementation aligned with the Comprehensive School Physical Activity Program framework.

Additional features that have been recognized as critical to successful development and implementation include: resource availability, appropriateness and acceptability of the program for the school, a supportive school climate, and training to develop the confidence of delivery agents; conversely, barriers include time constraints and difficulties related to program scheduling and coordination [42]. Thus, effective collaboration is needed between stakeholders who bring varied expertise (e.g., educators, health professionals, researchers, parents) to ensure contextual appropriateness of programs, increase acceptability, maximize impact and minimize the influence of barriers.

A systematic umbrella review by Abu‐Omar et al. [43] highlights that most school‐based physical activity programs are cost‐effective for children and adolescents, particularly multi‐component programs and those involving environmental strategies. Moreover, it is likely that the savings associated with increased physical activity among children outweigh the costs of program delivery [44]. Thus, greater resourcing priority should be given to scaling programs in settings such as schools that can have widespread health economic benefits.

Cross‐sector partnership between public organizations and private (both for‐profit and philanthropic) organizations, referred to as public‐private partnerships, offers promising solutions for implementing health programs at scale to pursue shared goals and achieve mutually beneficial outcomes [45]. Public‐private partnerships can help address health inequalities by adopting complementary actions that span from a multi‐tiered support system in the school context, where all children and adolescents can be reached, to clinical contexts tailor‐made for special populations. In public‐private partnerships, researchers and community partners collaborate to conduct rigorous research, extend evidence‐based interventions across multiple settings, and sustain their implementation and impact over time. This approach is referred to as a dual‐logic framework [46]. The dual‐logic framework recognizes that two distinct but complementary systems must operate in tandem: Research logic, which prioritizes scientific evidence, intervention fidelity, and theory‐driven design, and scale‐up logic, which emphasizes population reach, implementation timeliness, and long‐term sustainability within real‐world systems.

To support program development, implementation and sustainability, charities and industries can play a key role in adapting evidence‐based content into accessible, engaging, and contextually appropriate formats. This dual‐logic approach is operationalized, for example, in AllPlay Dance [47]. AllPlay Dance provides an inclusive teaching model to enable children of all abilities to experience community‐based dance. It brings together psychology, neuroscience, and arts disciplines to produce relevant content, whereas also partnering with creative industries including graphic designers and software engineers to develop program assets. AllPlay Dance, funded through philanthropic and government partnerships, embeds research rigour within implementation processes, providing an example of how research validity and practical scalability can be integrated rather than treated as competing priorities. Rather than conducting research and implementation as separate sequential phases, the dual‐logic framework positions them as simultaneous, mutually reinforcing processes that enhance both scientific understanding and real‐world impact.

In addition to philanthropic support, Corporate Social Responsibility initiatives play a crucial role within public‐private partnerships, ensuring programs move beyond initial adoption to sustained implementation. Corporate Social Responsibility, defined as voluntary corporate actions that promote social and environmental well‐being beyond regulatory requirements [48], provides critical support for the application, scalability and sustainability of evidence‐based interventions [49]. However, regarding the promotion of physical activity among children, Leone et al.'s analysis [14] discusses the risk of Corporate Social Responsibility serving corporate marketing goals under the guise of promoting children's wellbeing and emphasizes the critical role of good governance structures to mitigate potential risks. Key elements for promoting health interests include: aligning funding with children's rights frameworks and public health ethics; investing in long‐term funding and partnership strategies to avoid ‘tokenistic’ or opportunistic efforts; collaborating with the university sector to ensure evidence‐based scientific grounding; and working with government to ensure shared responsibility [14, 50].

Dual‐logic approaches offer an opportunity to more effectively bridge research and government priorities [45]. Such model combines the realities of applying scientific rigour and causal insight with the urgency of policy‐driven timeliness, equity and scalability, strengthening the translation of evidence into effective practice. Rather than progressing in a linear format from research to policy, the dual‐logic approach proposes integration of both from the outset, using hybrid designs, shared governance, and real‐time evaluation to enable adaptive, equitable and scalable implementation of programs.

## 5 Conclusions: Toward a Multifaceted Understanding of Holistic Physical Activity Promotion for Children and Adolescents

Physical activity represents one of the most accessible and modifiable factors capable of influencing holistic health and wellbeing, with benefits spanning physical, cognitive, emotional and social domains. When implemented in structured and engaging environments such as schools, physical activity programs can mitigate the effects of lifestyle risk factors that contribute to health inequities. Aligned with the World Health Organization's [7] model of health, there is a need for children to have greater access to programs that serve as a foundation for whole‐child development, connecting movement, learning and wellbeing within and beyond the school environment. This paper provides examples of global research programs that illustrate how evidence‐based, multisectoral, and transdisciplinary approaches to youth physical activity can foster equitable and scalable health promotion across educational and community settings. Leveraging Public‐Private Partnerships can promote greater collaboration among stakeholders, community engagement, and adequate resourcing and support for delivery organizations. In combination with hybrid designs, shared governance and real‐time evaluation, these approaches can facilitate adaptive, equitable and scalable implementation of physical activity initiatives for children and adolescents worldwide.

## Author Contributions

**Nicole Rinehart:** conceptualization, writing – original draft, writing – review and editing. **Carlo Agostoni:** writing – review and editing. **Chloe Emonson:** conceptualization, writing – original draft, writing – review and editing. **Caterina Pesce:** conceptualization, writing – original draft, writing – review and editing. **Livio Luzi:** writing – review and editing. **Spyridoula Vazou:** conceptualization, writing – original draft, writing – review and editing.

## Conflicts of Interest

N.R. has received research funding from Jonathan and Simone Wenig, the National Health and Medical Research Council, Adam Krongold, MECCA M‐Power, Victorian Department of Education, NSW Department of Education, Grace & Emilio Foundation, Moose Happy Kids Foundation, Department of Social Services—Information, Linkages and Capacity Building (ILC) Program, Soremartec (Ferrero Group) as part of its Kinder Joy of moving pillar of Corporate Social Responsibility initiatives, and Aspen Pharmacare Australia Pty Ltd. She has consulted or served on advisory boards for the Victorian Department of Education, the Melbourne Children's Clinic, and AMAZE (Autism Victoria). She has research partnerships with the Australian Football League, Queensland Ballet, the Victorian Department of Education, the NSW Department of Education, Beijing Dance Academy, and the Moose Happy Kids Foundation. C.E. has worked on projects with research funding from Jonathan and Simone Wenig, Adam Krongold, MECCA M‐Power, Victorian Department of Education, Grace & Emilio Foundation, Moose Happy Kids Foundation, Department of Social Services—Information, Linkages and Capacity Building (ILC) Program, Soremartec (Ferrero Group) as a part of its Kinder Joy of moving pillar of Corporate Social Responsibility initiatives, and Aspen Pharmacare Australia Pty Ltd. She works on research partnerships with the Australian Football League, Queensland Ballet, the Victorian Department of Education, the Beijing Dance Academy, and the Moose Happy Kids Foundation. C.P. has received honoraria from Soremartec for developing and monitoring the ‘Joy of Moving’ educational model and training teachers. However, her role in the research projects granted by the corporate and cited in this paper has not influenced the outcomes reported. C.A. is a Consultant of Soremartec. L.L. has been a Consultant of Soremartec during the last 2 years. S.V. has received funding from Ferrero USA Corporate Social Responsibility.

## Data Availability

The authors have nothing to report.
